# Network-based analysis of vaccine-related associations reveals consistent knowledge with the vaccine ontology

**DOI:** 10.1186/2041-1480-4-33

**Published:** 2013-11-11

**Authors:** Yuji Zhang, Cui Tao, Yongqun He, Pradip Kanjamala, Hongfang Liu

**Affiliations:** 1 Division of Biomedical Statistics and Informatics, Department of Health Sciences Research, Mayo Clinic, Rochester, MN 55905, USA; 2 School of Biomedical Informatics, University of Texas Health Science Center at Houston, Houston, TX 77030, USA; 3 Unit of Laboratory of Animal Medicine, University of Michigan, Ann Arbor, MI 48109, USA

## Abstract

**Background:**

Ontologies are useful in many branches of biomedical research. For instance, in the vaccine domain, the community-based Vaccine Ontology (VO) has been widely used to promote vaccine data standardization, integration, and computer-assisted reasoning. However, a major challenge in the VO has been to construct ontologies of vaccine functions, given incomplete vaccine knowledge and inconsistencies in how this knowledge is manually curated.

**Results:**

In this study, we show that network-based analysis of vaccine-related networks can identify underlying structural information consistent with that captured by the VO, and commonalities in the vaccine adverse events for vaccines and for diseases to produce new hypotheses about pathomechanisms involving the vaccine and the disease status. First, a vaccine-vaccine network was inferred by applying a bipartite network projection strategy to the vaccine-disease network extracted from the Semantic MEDLINE database. In total, 76 vaccines and 573 relationships were identified to construct the vaccine network. The shortest paths between all pairs of vaccines were calculated within the vaccine network. The correlation between the shortest paths of vaccine pairs and their semantic similarities in the VO was then investigated. Second, a vaccine-gene network was also constructed. In this network, 4 genes were identified as hubs interacting with at least 3 vaccines, and 4 vaccines were identified as hubs associated with at least 3 genes. These findings correlate with existing knowledge and provide new hypotheses in the fundamental interaction mechanisms involving vaccines, diseases, and genes.

**Conclusions:**

In this study, we demonstrated that a combinatorial analysis using a literature knowledgebase, semantic technology, and ontology is able to reveal important unidentified knowledge critical to biomedical research and public health and to generate testable hypotheses for future experimental verification. As the associations from Semantic MEDLINE remain incomplete, we expect to extend this work by (1) integrating additional association databases to complement Semantic MEDLINE knowledge, (2) extending the neighbor genes of vaccine-associated genes, and (3) assigning confidence weights to different types of associations or associations from different sources.

## Background

Vaccines have been one of the most successful public health interventions to date, with most vaccine-preventable diseases having declined in the United States by 95% to 99% [1]. However, vaccine development has become more difficult as more complex organisms become vaccine targets. In recent years, drug repositioning has increased rapidly and achieved a number of successes for existing drugs, such as sildenafil [2] and thalidomide [3]. By definition, drug repositioning is the “process of finding new uses outside the scope of the original medical indications for existing drugs or compounds” [4]. In 2009, more than 30% of 51 new medicines and vaccines were developed on the basis of previously marketed products. This suggests that drug repositioning has drawn great attention from both industry and academic institutions [5]. However, many drug repositioning examples were based on serendipitous discoveries [6] or on observable clinical phenotypes, without systematic identification of new targets. Recent research has shown that bioinformatics-based approaches can aid in repositioning drugs based on the complex relationships among drugs, diseases, and genes [7].

In recent years, high-throughput biological data and computational systems biology approaches have provided an unprecedented opportunity to understand disease etiology and its underlying cellular subsystems. Biological knowledge, such as drug-disease networks and biomedical ontologies, have accelerated the development of network-based approaches to understanding disease etiologies [8,9] and drug actions (network pharmacology) [10,11]. Such approaches could also be applied to vaccine research, aiming to investigate vaccine-related associations derived from public knowledgebases, such as PubMed. For example, Vaccine Ontology (VO)–based literature mining research last year studied all potential gene interactions associated with fever alone or both fever and vaccine [12]. This study focused on the retrieval of gene-gene associations identified on the basis of their direct interactions in the context of fever and vaccine. The centrality-based network approach [13] evaluated the level of importance for each gene in the extracted gene interaction network. Novel gene interactions were identified to be essential in fever- or vaccine-related networks that could not be found before. A similar VO and centrality-based literature mining approach was used to analyze a vaccine-associated interferon γ gene interaction network [14].

Ball et al. [15] compiled a network consisting of 6,428 nodes (74 vaccines and 6,354 adverse events) and more than 1.4 million interlinkages, derived from the Vaccine Adverse Event Reporting System. This network demonstrated a scale-free property, in which certain vaccines and adverse events acted as “hubs.” Such network analysis approaches complement current statistical techniques by offering a novel way to visualize and evaluate vaccine adverse event data. However, the relationships among different vaccines in the context of vaccine-vaccine and vaccine-gene networks have not been well studied. A systematic investigation of such relationships will improve understanding of how vaccines are related to each other and whether such information can complement existing knowledge, such as the VO, therefore providing possible directions for future drug-repositioning.

To analyze the possible commonly shared protective immunity or adverse event mechanisms among different vaccines, it is critical to study all possible vaccine-vaccine and vaccine-gene associations using network analysis approaches. The hypotheses behind this are 2-fold: (1) if 2 vaccines have a coupling relationship with common disease(s) or gene(s), they are linked in the vaccine network; and (2) the closer 2 vaccines are in the vaccine network, the more similar they are in the context of a literature knowledgebase, such as Semantic MEDLINE [16]. In this study, we proposed a network-based approach to investigate the underlying relationships among vaccines in the context of the vaccine-related network derived from Semantic MEDLINE. The distances of the vaccines were further compared with their semantic similarities in the VO. The results demonstrated that the structure information in the vaccine network is consistent with that captured by the VO. Such network-based analysis can serve as an independent data resource to construct and evaluate biomedical ontologies. In addition, the vaccine-gene network was constructed also on the basis of Semantic MEDLINE information, in which important vaccine-related genes were identified and investigated by the VO and related independent resources. Both analyses demonstrated the potential to serve as the basis for future drug discovery and repositioning. The results of this study will establish associations that may kindle hypothesis generation about drug repositioning.

The rest of the paper is organized as follows. Section 2 introduces the data resources and the proposed network-based framework. Section 3 illustrates the results generated from each step in the proposed computational framework. Section 4 provides a thorough discussion of the results and concludes the paper.

## Materials and methods

**Figure 1:**
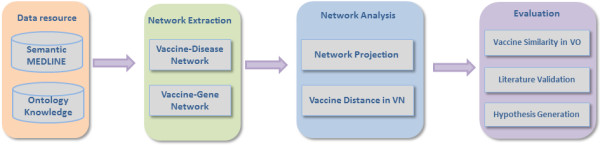
**Overview of the proposed framework.** The proposed method consists of 3 steps: (1) extraction of vaccine-related associations from Semantic MEDLINE using ontology-based terms; (2) network-based analyses to identify vaccine-vaccine associations and vaccine-gene associations; and (3) evaluation of inferred vaccine-vaccine and vaccine-gene relationships using VO hierarchical structure and literature validation.

In this section, we first describe the data resources and preprocessing method in this work. We then introduce our proposed network-based approach for investigating vaccine-related associations derived from literature abstracts in PubMed. The evaluation of the discovered vaccine-vaccine and vaccine-gene relationships is based on the VO hierarchy and logical definitions. Figure 1 illustrates the steps of the proposed approach.

### Data sources and preprocessing

#### Data resources

In this study, we used Semantic MEDLINE as the data resource to build the networks. Semantic MEDLINE [16] is a National Library of Medicine–initiated project, which provides a publicly available database that contains comprehensive resources, with structured annotations for information extracted from more than 19 million MEDLINE abstracts. Since Semantic MEDLINE is a comprehensive resource that contains heterogeneous data with different features extracted, our previous research has reorganized this data source and optimized it for informatics analysis [17]. Using the Unified Medical Language System semantic types and groups [18], we extracted unique associations among diseases, genes, and drugs and represented them in 6 Resource Description Framework (RDF) graphs. In this study, we used our optimized Semantic MEDLINE RDF data as the data source to perform network analysis for vaccine-related networks.

Our RDF-based Semantic MEDLINE resource currently contains 843,000 disease-disease, 111,000 disease-gene, 1,277,000 disease-drug, 248,000 drug-gene, 1,900,000 drug-drug, and 49,000 gene-gene associations. Since this resource contains high-level terms (eg, *gene*, *protein*, *disease*) that are not useful for network analysis, we manually filtered out these terms. For disease terms, we used only those terms that are included in International Classification of Diseases, Ninth Revision (ICD 9). For gene terms, we included only those terms that have an Entrez Gene ID.

#### Data extraction

We identified the associations relevant to vaccines only. Specifically, vaccine terms were identified on the basis of Systematized Nomenclature of Medicine–Clinical Terms (SNOMED CT) (http://www.ihtsdo.org/snomed-ct). All the terms under the SNOMED CT term *Vaccine* (CUI: C0042210) were first extracted. Manual review by 3 experts further removed common terms (eg, *bacteria*, *vaccine*) and animal vaccine terms.

### Network analysis of vaccine network

#### Projection of bipartite vaccine-disease network

In graph theory, a bipartite network is composed of 2 non-overlapping sets of nodes and links that connect 1 node in the first node set with 1 node in the second node set. The properties of bipartite networks are often investigated by considering the 1-mode projection of the bipartite network. The 1-mode projection network can be created by connecting 2 nodes in the same node set if they have at least 1 common neighboring node in the other node set. For instance, the vaccine-disease association network is 1 bipartite network: vaccines and diseases constitute 2 node sets, and links are generated between vaccine and disease if they are associated in the Semantic MEDLINE. Therefore, the vaccine-vaccine network can be investigated by projecting vaccine-disease associations to vaccine-vaccine associations, in which 2 vaccines are connected if they are associated with at least 1 same disease. In this work, all links were generated on the basis of associations extracted from Semantic MEDLINE as described in the preceding section, “Data Sources and Preprocessing.” A vaccine-vaccine network was generated consisting of all the links identified in vaccine-disease associations.

### Network distance between vaccines

The distance between any two vaccines in the vaccine network was calculated as the length of the shortest path between them [19]. The hierarchical clustering analysis was performed on the distance matrix of all vaccines [20]. Compared to other clustering methods (e.g., k-mean clustering), hhierarchical clustering doesn’t need specify the number of clusters in advance, generates small clusters that are more biologically meaningful, and produces a bottom-up hierarchical structure that is indicative of similarities among variables.

A heatmap is an effective way to visually display multivariate data, which combines the use of color to distinguish the magnitude of measurements and dendrograms to show clustering of variables [21]. Heatmaps are often used for gene expression data to identify the similairty among genes or samples. In this study, we used heatmaps to investigate the hierarchical associations among vaccine terms.

### Analysis of vaccine-gene network

The vaccine-gene network was constructed by vaccine-gene associations extracted from the drug-gene associations in our RDF-based data resource. The important vaccine-related genes were identified by their significant higher node degree compared with other vaccines or genes in the same network. The Cytoscape tool [22] was used to visualize the network. Cytoscape is an open-source platform for integration, visualization, and analysis of biological networks. Its functionalities can be extended through Cytoscape plugins. Scientists from different research fields have contributed more than 160 useful plugins so far. These comprehensive features allow us to perform thorough network-level analyses, visualization of our association tables, and integration with other biological networks in the future.

### Analysis of vaccine groups using VO

The community-based VO includes more than 4,000 vaccine-specific terms, namely, all licensed human and veterinary vaccines currently used in the United States. Logical axioms have been defined in the VO to represent the relations among vaccine terms [14]. The Semantic MEDLINE analysis uses SNOMED terms to represent various vaccines. The VO has established automatic mapping between SNOMED vaccine terms and VO terms. On the basis of the mapping, we first extracted all vaccine terms from the Semantic MEDLINE and mapped to the VO. The ontology term retrieval tool OntoFox [23] was then applied to obtain the hierarchies of the total vaccines or subgroups of the vaccines identified in this study.

## Results

### The overall network view

In total, 76 vaccines, annotated by the SNOMED CT term *Vaccine* (CUI: C0042210), were used to extract related vaccine-disease and vaccine-gene associations from the drug-disease and drug-gene association tables, respectively. In the vaccine-disease network, there were 1,038 nodes (104 vaccines and 934 diseases) and 1,693 vaccine-disease associations (Additional file 1). In the vaccine-gene network, there were 170 nodes (85 vaccines and 85 genes) (Additional file 2) and 94 vaccine-gene associations. One vaccine network was generated by the projection of the vaccine-disease bipartite network, consisting of 76 vaccines and 573 associations (Additional file 3). This vaccine network was then used to analyze the vaccine relationships. The derived vaccine-gene network was also investigated by the VO knowledge.

### Analysis of vaccine network

**Figure 2:**
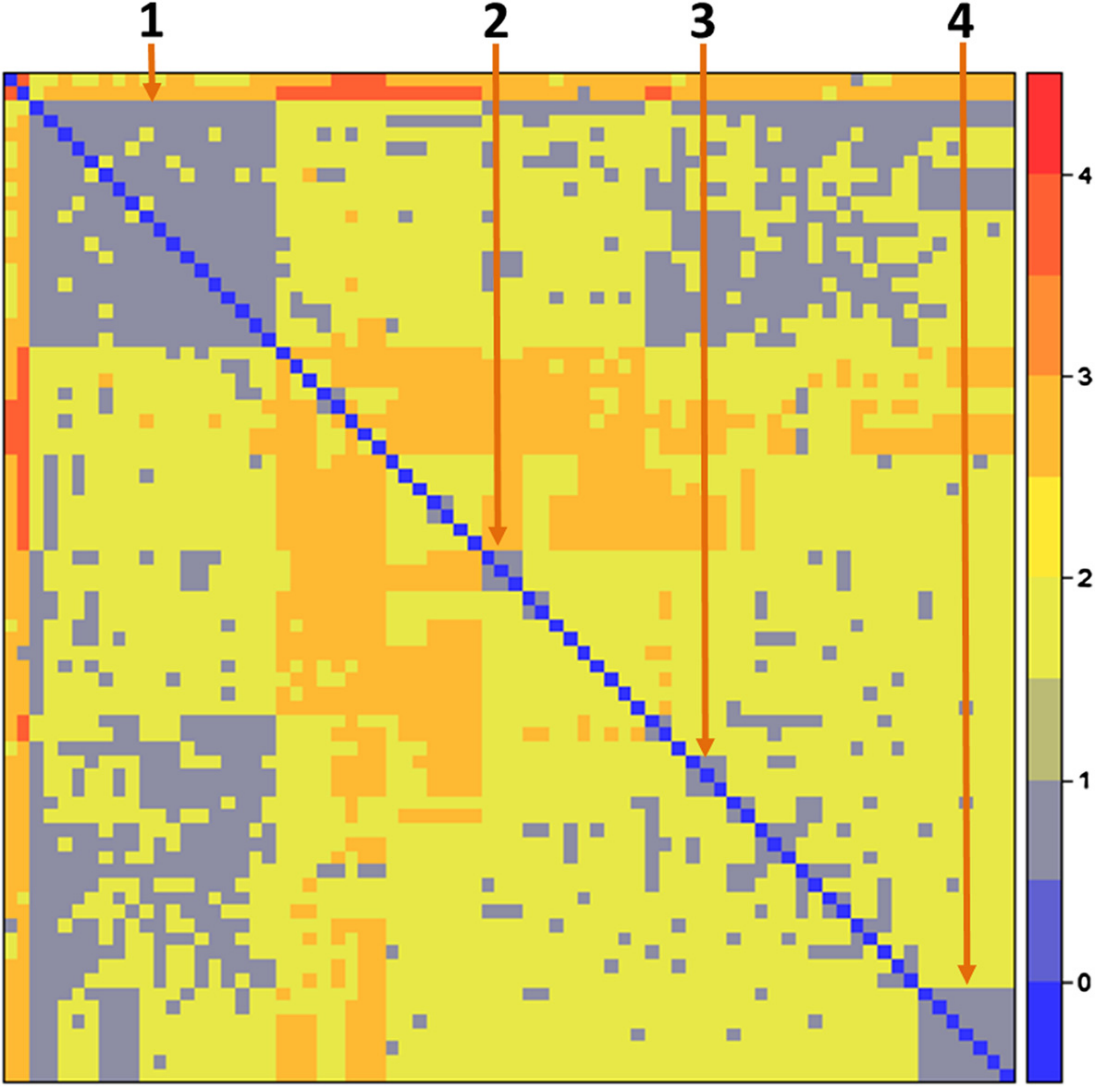
**The heat map of vaccine-vaccine associations.** The shortest path matrix of all vaccine pairs was used to generate the heat map. Each row (column) represents a vaccine term. The color scale represents the shortest path between any vaccine pair.

Figure 2 shows a heat map of hierarchical analysis results, providing direct visualization of potential vaccine-vaccine associations. Here we selected 4 relatively large vaccine-vaccine association groups on the diagonal from Figure 2 and explain them in detail:

#### Vaccine-vaccine association 1

**Figure 3:**
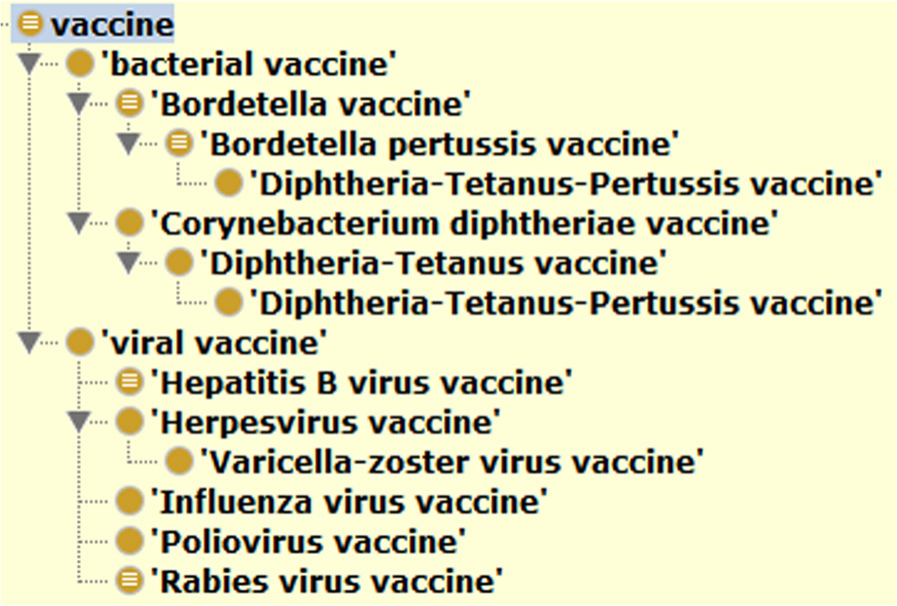
**The VO hierarchical structure of the seven vaccines associating with neurological disorder.** A reasoning process assigned the Diphtheria-Tetanus-Pertussis vaccine under *Bordetella pertussis* vaccine. The Protégé-OWL editor 4.2 was used for the figure generation.

This group contains 18 widely studied vaccines. Many interesting results have been obtained from the analysis of this group of vaccine-disease-vaccine associations. For example, the results from this group show that influenza vaccines and rabies vaccines have been associated with the induction of a severe adverse event, Guillain-Barré syndrome [24,25]. Guillain-Barré syndrome is a rare disorder in which a person’s own immune system damages the nerve cells, causing muscle weakness and sometimes paralysis. This group also includes 5 other vaccines associated with nervous system disorders, including pertussis vaccine [26], diphtheria and tetanus toxoids and pertussis (DTP) vaccine [27], hepatitis B vaccine [28], chickenpox vaccine [29], and poliovirus vaccine [30,31]. As shown by a VO hierarchical structure layout (Figure 3), these 7 vaccines belong to different bacterial and viral vaccine groups. For instance, the DTP vaccine is a combination vaccine that contains 3 individual vaccine components, including a pertussis vaccine. DTP is asserted in the VO as a subclass of diphtheria and tetanus toxoids vaccine. In SNOMED, DTP is asserted as subclass of a “Diphteria + tetanus vaccine”, which is a subclass of 'tetanus vaccine’ and 'diphtheria vaccine’. However, the DTP term in SNOMED does not include any axiom that makes the association of the vaccine to any of the pathogens that causes the three diseases. Different from SNOMED, the VO logically defines vaccines by associating each vaccine to the pathogen organisms defined in the NCBI Taxonomy Ontology. For example, a VO vaccine for diphtheria has the following axiom definition: *'vaccine immunization against microbe’ some 'Corynebacterium diphtheriae’*. This logical definition links the VO vaccine to the bacterium '*Corynebacterium diphtheriae*’ defined in the NCBOTaxon ontology with the ID: NCBITaxon_1717. Since multiple inheritances are not used in the VO, an inference applying an ontology reasoner was used to infer that the DTP is also *Bordetella pertussis* vaccine (ie, pertussis vaccine) (Figure 3). It is likely that the association of the combination vaccine DTP with a neurologic disorder is at least partially attributable to the pertussis vaccine component.

Our study also identified many other diseases associated with different vaccines. For example, 5 vaccines (eg, pertussis vaccine) were found to be associated with various types of antimicrobial susceptibility, and 8 vaccines (eg, influenza vaccine) have been co-studied in patients with the asthma condition. Because of the relatively poor annotation of the vaccine data in the Semantic MEDLINE system, the vaccines identified in the semantic analysis were poorly classified. Our previous study [32] also confirmed that the vaccine annotations in VO are more granular than that in Semantic MEDLINE or MeSH. The incorporation of the VO in the study clearly classifies these vaccines, leading to better understanding of the result of the Semantic MEDLINE analysis.

#### Vaccine-vaccine association 2

This group of vaccines, including Q fever vaccine, parvovirus vaccine, and tick-borne encephalitis vaccine, is associated with the common disease delayed hypersensitivity. Delayed-type reactions may occur at days after vaccination and often raise serious safety concerns. Delayed hypersensitivity is not antibody mediated but rather is a type of cell-mediated response. The study of common vaccines and gene and pathway features related to the delayed reaction will help to reveal the cause of a delayed-type reaction and eventually prevent it. While these vaccines are developed against different bacterial or viral diseases, there may be similarities among the vaccines, such as common vaccine ingredients (eg, adjuvant) and a shared target in some common biological pathway in human. Identification of these common features may indicate a common cause of a delayed-type reaction.

#### Vaccine-vaccine association 3

This group of vaccines is associated with the common disease mumps. The vaccines in this group include mumps vaccine, measles, mumps, rubella, and varicella virus vaccine, and DTP-*Haemophilus* b conjugate vaccine (DTP-Hib). The first 2 vaccines protect against mumps. The DTP-Hib vaccine was compared with a mumps vaccine in a study [33].

#### Vaccine-vaccine association 4

This vaccine group consists of 7 vaccines (eg, *Brucella abortus* vaccine and bovine rhinotracheitis vaccine) with direct associations between them. They are all associated with the common term *calf* in the literature abstracts. Since calf disease is a synonym Scheuermann disease, these vaccines have all been linked to Scheuermann disease because of the ambiguity of the Natural Language Processing (NLP) process. This ambiguity can be improved by future advancement in the disambiguity capacity of the NLP tools.

### Vaccine-gene network

**Figure 4:**
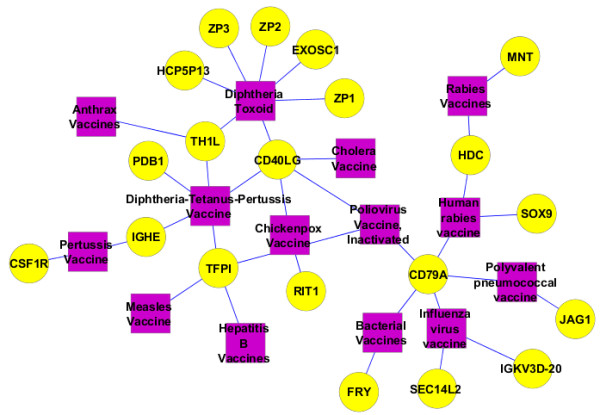
**A vaccine-gene subnetwork.** The associations between vaccines and related genes were visualized by the Cytoscape tool [22]. Rectangular purple nodes represent vaccines, and round yellow nodes represent genes.

In the vaccine-gene network (Additional file 4), many genes were found to interact with different vaccines. Four genes (*TH1L*, *CD40LG*, *TFPI*, and *CD79A*) have been identified as interacting with at least 3 vaccines. These 4 genes can be considered hub genes in the vaccine-gene network. Among them, *CD40LG* (CD40 ligand) is closely associated with 5 vaccines: diphtheria toxoid vaccine, cholera vaccine, tetanus toxoid vaccine, chickenpox vaccine, and inactivated poliovirus vaccine (Figure 4). *CD40LG* plays an important role in antigen presentation and stimulation of cytotoxic T lymphocytes [34]. *CD40LG* can also be used in rational vaccine adjuvant design [35]. It is likely that all 5 of these vaccines stimulate protective immunity through the same *CD40LG*-mediated pathway. Our finding confirms the important role of *CD40LG* and provides a possible hypothesis on how different bacterial and viral vaccines stimulate a protective immune response in the host. Another example is *CD79A* (immunoglobulin-associated alpha, also known as mb-1), a phosphoprotein that is a component of the CD79a/CD79b dimer associated with membrane-bound immunoglobulin in B cells. This dimer interacts with the B-cell antigen receptor and enables the cell to respond to the antigens on the cell surface [36]. The presence of the CD79a suggests strong B-cell–mediated antibody response. Interestingly, both *CD40LG* and *CD79A* interact with the same inactivated poliovirus vaccine. It is likely that the inactivated poliovirus vaccine induces both *CD40LG*-mediated cellular immune response and *CD79A*-mediated B-cell antibody response.

In the gene-vaccine network, many vaccines also interact with multiple genes. Four vaccines (diphtheria toxoid vaccine, DTP vaccine, inactivated poliovirus vaccine, and influenza virus vaccine) were found to interact with at least 3 genes. According to the VO, diphtheria toxoid vaccine protects against infection by *Corynebacterium diphtheriae*. In addition to *C diphtheriae*, the DTP vaccine also protects against infection by *Clostridium tenani* and *Bordetella pertussis*. Both of these vaccines interact with *CD40LG* and *TH1L*. So it is likely that these 2 proteins specifically interact with the same *C diphtheriae* component in diphtheria toxoid vaccine, but not with the other components in the DTP vaccine.

### Conclusions and future work

In this paper, we proposed a novel network-based approach to investigate vaccine relationships in the context of a vaccine network extracted from abstracts from the literature posted to PubMed. These investigations of vaccine-vaccine, vaccine-disease, and vaccine-gene networks demonstrated that such literature-based associations can be better analyzed using the VO and such a combinatorial analysis is able to reveal the association patterns and new knowledge. The identified vaccine-vaccine associations based on vaccine-disease distance analysis were consistent with their VO categories and often lead to the generation of new hypotheses. Our studies identified some novel vaccine-vaccine relationships by discovering a group of vaccines associated with some common diseases, as demonstrated in the heat map analysis in the Results section. Because information in the literature is incomplete, such vaccine-vaccine associations need further validation in independent databases or through future experimental studies. For example, while our analysis revealed associations between a group of vaccines and neurologic adverse events, the evidence of these associations, although reported by some PubMed abstracts, has not necessarily been commonly acknowledged [37]. More analysis may be required for clarification. As such relationships are further validated by independent data resource such as Electronic Health Record (EHR), they can complement current relationships in VO and provide additional underlying targets for vaccine repositioning in the future.

Extensions of this work include the following: (1) integration of more comprehensive vaccine-disease association databases (e.g., the Vaccine Adverse Event Reporting System) to construct more complete vaccine-related networks; (2) generation of a vaccine-related gene network by extending analysis to the neighbor genes of vaccine-associated genes; (3) network-based investigation of the relationships among vaccines and other drugs using vaccine-drug associations; and (4) investigation of possible ways to improve the network by assigning metrics such as weights or confidence rates to different types of associations or associations from different sources.

## Competing interests

The authors declare that they have no competing interests.

## Authors’ contributions

YZ and CT led the study design and analysis, and drafted the manuscript. YH contributed to the analysis of VO. YH and HL provided institutional support and manuscript editing. All authors read and approved the final manuscript.

## Supplementary Material

Additional file 1 The vaccine-disease network consisting of 1,038 nodes (104 vaccines and 934 diseases) and 1,693 vaccine-disease associations. Click here for file

Additional file 2 The vaccine-gene network consisting of 170 nodes (85 vaccines and 85 genes) and 94 vaccine-gene associations. Click here for file

Additional file 3 The vaccine network consisting of 76 vaccines and 573 associations. Click here for file

Additional file 4 The cys file (Cytoscape format) of the vaccine-gene network. Click here for file

## References

[B1] Prevention, C.f.D.C.a Reported vaccine-preventable diseases--United States, 1993, and the childhood immunization initiative MMWR Morb Mortal Wkly Rep 1994 43 4 57 60 8295625

[B2] Goldstein I Oral sildenafil in the treatment of erectile dysfunction. Sildenafil study group N Engl J Med 1998 338 20 1397 1404 9580646 10.1056/NEJM199805143382001

[B3] Singhal S Antitumor activity of thalidomide in refractory multiple myeloma N Engl J Med 1999 341 21 1565 1571 10564685 10.1056/NEJM199911183412102

[B4] Chong CR Sullivan DJ Jr New uses for old drugs Nature 2007 448 7154 645 646 17687303 10.1038/448645a

[B5] Graul AI The year’s new drugs & biologics - 2009 Drug News Perspect 2010 23 1 7 36 20155217 10.1358/dnp.2010.23.1.1440373

[B6] Ashburn TT Thor KB Drug repositioning: identifying and developing new uses for existing drugs Nat Rev Drug Discov 2004 3 8 673 683 15286734 10.1038/nrd1468

[B7] Liu Z In silico drug repositioning: what we need to know Drug Discov Today 2013 18 3–4 110 115 22935104 10.1016/j.drudis.2012.08.005

[B8] Ideker T Sharan R Protein networks in disease Genome Res 2008 18 4 644 652 18381899 10.1101/gr.071852.107 PMC3863981

[B9] Barabasi AL Gulbahce N Loscalzo J Network medicine: a network-based approach to human disease Nat Rev Genet 2011 12 1 56 68 21164525 10.1038/nrg2918 PMC3140052

[B10] Berger SI Iyengar R Network analyses in systems pharmacology Bioinformatics 2009 25 19 2466 2472 19648136 10.1093/bioinformatics/btp465 PMC2752618

[B11] Mathur S Dinakarpandian D Drug repositioning using disease associated biological processes and network analysis of drug targets AMIA Annu Symp Proc 2011 2011 305 311 22195082 PMC3243256

[B12] Hur J Identification of fever and vaccine-associated gene interaction networks using ontology-based literature mining J Biomed Semantics 2012 3 1 18 23256563 10.1186/2041-1480-3-18 PMC3599673

[B13] Ozgur A Identifying gene-disease associations using centrality on a literature mined gene-interaction network Bioinformatics 2008 24 13 i277 i285 18586725 10.1093/bioinformatics/btn182 PMC2718658

[B14] Ozgur A Mining of vaccine-associated IFN-gamma gene interaction networks using the vaccine ontology J Biomed Semantics 2011 2 Suppl 2 S8 21624163 10.1186/2041-1480-2-S2-S8 PMC3102897

[B15] Ball R Botsis T Can network analysis improve pattern recognition among adverse events following immunization reported to VAERS? Clin Pharmacol Ther 2011 90 2 271 278 21677640 10.1038/clpt.2011.119

[B16] Rindflesch TC Semantic MEDLINE: an advanced information management application for biomedicine Inf Serv & Use 2011 31 1/2 15 21

[B17] Tao C Optimizing Semantic MEDLINE for Translational Science Studies Using Semantic Web Technologies Proceedings of the 2nd international workshop on Managing interoperability and compleXity in health systems2012 2012 Maui, Hawaii, USA: ACM 53 58

[B18] The UMLS Semantic Groups 2012 http://semanticnetwork.nlm.nih.gov/SemGroups/

[B19] Fekete A Vattay G Posfai M Shortest path discovery of complex networks Phys Rev E Stat Nonlin Soft Matter Phys 2009 79 065101 19658546 10.1103/PhysRevE.79.065101

[B20] Guess MJ Wilson SB Introduction to hierarchical clustering J Clin Neurophysiol 2002 19 2 144 151 11997725 10.1097/00004691-200203000-00005

[B21] Wilkinson L Friendly M The history of the cluster heat map Am Stat 2009 63 2 179 184

[B22] Smoot ME Cytoscape 2.8: new features for data integration and network visualization Bioinformatics 2011 27 3 431 432 21149340 10.1093/bioinformatics/btq675 PMC3031041

[B23] Xiang Z OntoFox: web-based support for ontology reuse BMC research notes 2010 3 175 20569493 10.1186/1756-0500-3-175 PMC2911465

[B24] Hartung HP [Guillain-Barre syndrome after exposure to influenza] Nervenarzt 2012 83 6 714 730 22528062 10.1007/s00115-012-3479-8

[B25] Hemachudha T Immunologic studies of rabies vaccination-induced Guillain-Barre syndrome Neurology 1988 38 3 375 378 2450302 10.1212/wnl.38.3.375

[B26] Wardlaw AC Animal models for pertussis vaccine neurotoxicity Tokai J Exp Clin Med 1988 13 Suppl 171 175 3078805

[B27] Corkins M Grose C Halbur T Fatal pertussis in an Iowa infant Iowa Med 1991 81 9 383 384 1743930

[B28] Comenge Y Girard M Multiple sclerosis and hepatitis B vaccination: adding the credibility of molecular biology to an unusual level of clinical and epidemiological evidence Med Hypotheses 2006 66 1 84 86 16176857 10.1016/j.mehy.2005.08.012

[B29] Bozzola E Neurological complications of varicella in childhood: case series and a systematic review of the literature Vaccine 2012 30 39 5785 5790 22683522 10.1016/j.vaccine.2012.05.057

[B30] Friedrich F Neurologic complications associated with oral poliovirus vaccine and genomic variability of the vaccine strains after multiplication in humans Acta virologica 1998 42 3 187 194 9842449

[B31] Korsun N Three cases of paralytic poliomyelitis associated with type 3 vaccine poliovirus strains in Bulgaria J Med Virol 2009 81 9 1661 1667 19626606 10.1002/jmv.21545

[B32] Hur J Ontology-based Brucella vaccine literature indexing and systematic analysis of gene-vaccine association network BMC immunology 2011 12 49 21871085 10.1186/1471-2172-12-49 PMC3180695

[B33] Henderson R General practitioners’ concerns about childhood immunisation and suggestions for improving professional support and vaccine uptake Commun Dis Public Health 2004 7 4 260 266 15779786

[B34] Kornbluth RS The emerging role of CD40 ligand in HIV infection J Leukoc Biol 2000 68 3 373 382 10985254

[B35] Kornbluth RS Stone GW Immunostimulatory combinations: designing the next generation of vaccine adjuvants J Leukoc Biol 2006 80 5 1084 1102 16931603 10.1189/jlb.0306147

[B36] Herren B Burrows PD B cell-restricted human mb-1 gene: expression, function, and lineage infidelity Immunol Res 2002 26 1–3 35 43 12403343 10.1385/ir:26:1-3:035

[B37] Sarntivijai S Ontology-based combinatorial comparative analysis of adverse events associated with killed and live influenza vaccines PLoS One 2012 7 11 e49941 23209624 10.1371/journal.pone.0049941 PMC3509157

